# Imaging of a Cilioretinal Artery Embolisation

**DOI:** 10.3390/ijms150915734

**Published:** 2014-09-04

**Authors:** Marion R. Munk, Rukhsana G. Mirza, Lee M. Jampol

**Affiliations:** 1 Department of Ophthalmology, Northwestern University, Feinberg School of Medicine, Chicago, IL 60611, USA; E-Mails: marion.munk@northwestern.edu (M.R.M.); r-mirza@northwestern.edu (R.G.M.); 2 Department of Ophthalmology, Medical University of Vienna, Vienna 1090, Austria

**Keywords:** retinal artery occlusion, spectral domain optical coherence tomography (SD-OCT), fundus autofluorescence, retinal ischemia, endarterectomy, cardiovascular disorder, atherosclerosis

## Abstract

Retinal artery occlusion can be the first indicator of a significant cardiovascular disorder and the need for treatment. We present the case of a 69-year-old man with a cilioretinal artery occlusion and retinal ischemia. Retinal imaging, in particular fundus autofluorescence, highlighted an intraluminal hyperautofluorescent lesion which led to the diagnosis of retinal emboli. Subsequently a severe, previously undiagnosed carotid occlusive disease was discovered. The patient underwent prompt endarterectomy.

## 1. Introduction

Retinal artery occlusion and retinal ischemia can be the first signs of a significant and symptomatic cardiovascular disorder and indicators of an urgent need for treatment. We show a case of a patient who presented with visual complaints and was found to have two focal areas of retinal whitening. Due to a complicated systemic history, his presentation led initially to a workup by the urgent care clinic for infectious retinitis and septic emboli. However, after exclusion of an infectious origin, the intraluminal hyperautofluorescent plaques in the macular arterioles were detected on retinal imaging, which led to the correct diagnosis of retinal emboli and associated retinal ischemia. These specific features led to the finding of severe, previously undiagnosed carotid occlusive disease. The patient underwent prompt endarterectomy, thus preventing further events.

## 2. Case Report

A 69-year-old male awoke with a “foggy spot the size of a pencil eraser” in the center of his vision of the right eye. His ocular history was unremarkable. His medical history was notable for hypertension and hyperlipidemia, which were treated with Amlodipine 5 mg and Simvastatin 40 mg once a day. He admitted ethanol abuse (about 1 bottle of bourbon per day). In addition, he reported a recent episode of cough and night sweats. He also complained of green sputum and chills.

**Figure 1:**
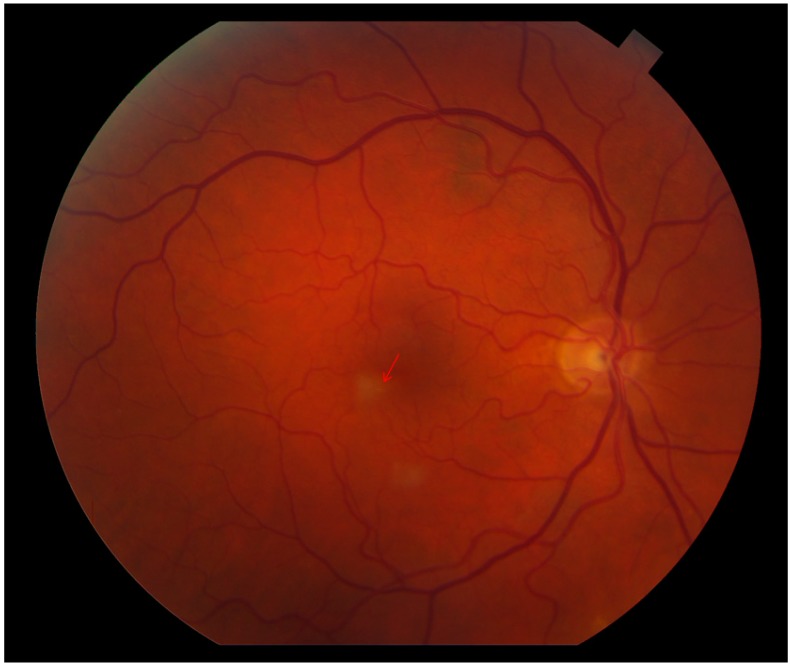
Color fundus image OD reveals two retinal parafoveal whitish lesions. The tiny, yellowish emboli in the small afferent arterioles are obscured by the retinal whitening (arrow).

**Figure 2:**
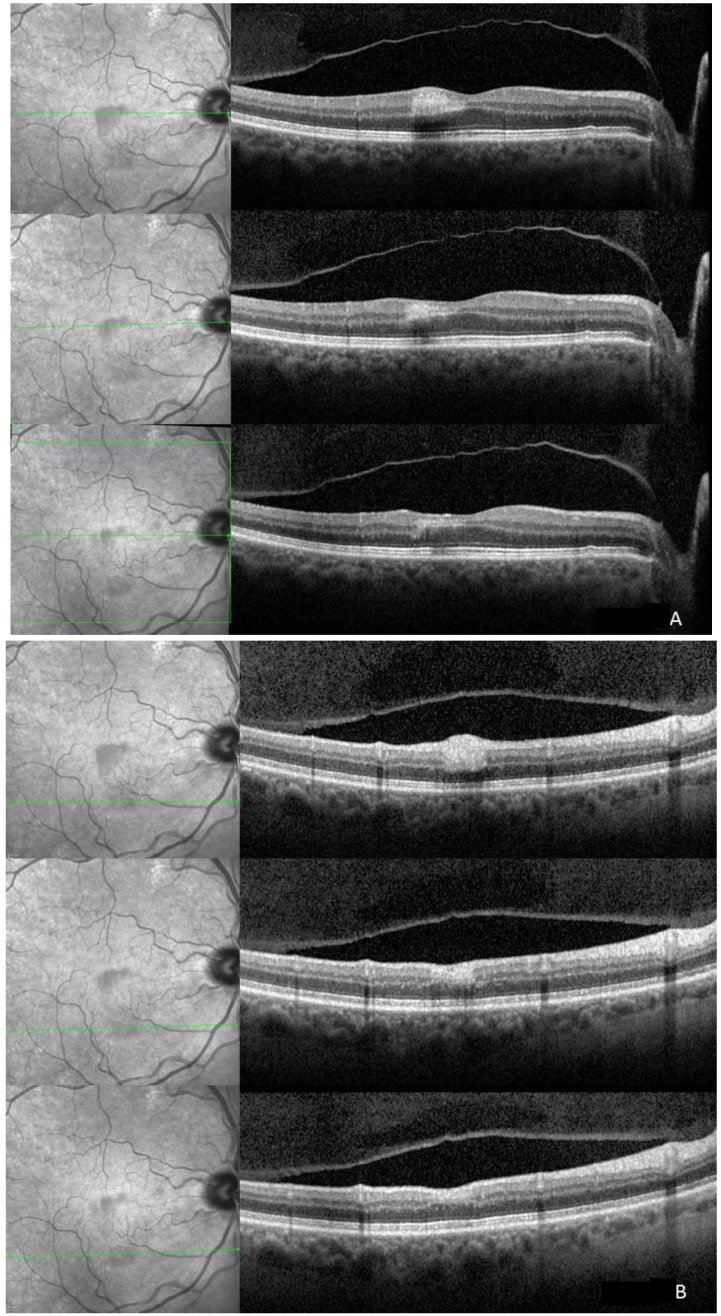
**Top**: The ischemic superior (**A**) and inferior (**B**) lesions show corresponding hyperreflectivity in the outer plexiform (OPL), inner nuclear (INL), inner plexiform (IPL), ganglion cell (GCL), and retinal nerve fiber layer (RNFL) on spectral domain optical coherence tomography (SD-OCT). On infrared image the ischemic areas are hyporeflective due to the scattering and/or absorbance of light. The four hyperreflective bands corresponding to the retinal pigment epithelium and the photoreceptors seem intact; **Middle**: Two weeks after initial presentation the hyperreflectivity is still visible and thinning of the respective layers is already appreciable. Hyporeflectivity already starts to fade on infrared imaging; **Bottom**: One month after initial presentation hyperreflectivity on infrared is still present. Affected layers revealed further thinning and the inner nuclear layer is not identifiable any more.

**Figure 3:**
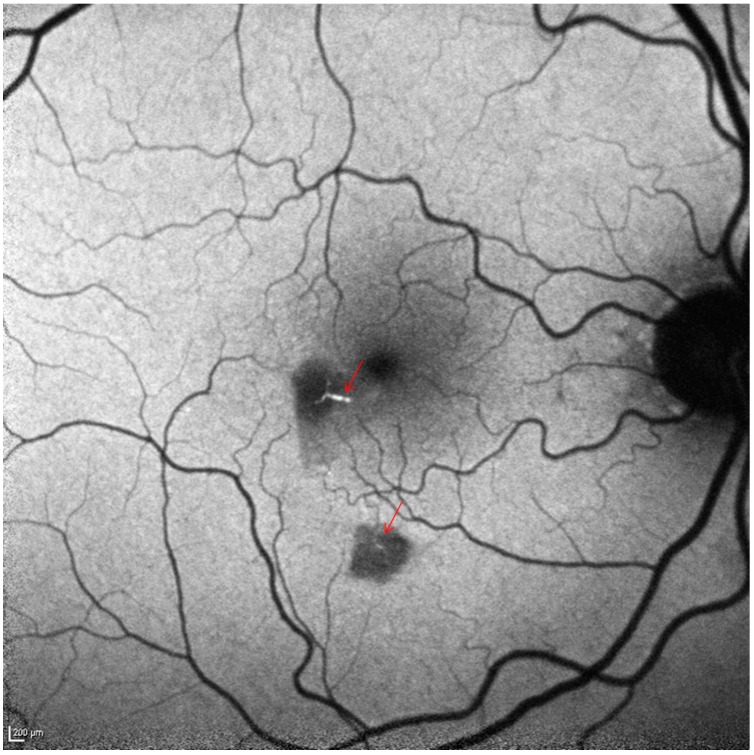
Short wave fundus autofluorescence shows two hypoautofluorescent areas (scattered and/or absorbed light due to swollen inner retina). The hyperautofluorescent emboli in the afferent arterioles are clearly visible (arrows). Scale bar: 200 µm.

At presentation to the urgent care clinic, his best corrected visual acuity was 20/20 OU and the anterior segment was unremarkable. Ophthalmoscopy revealed two parafoveal white retinal lesions (Figure 1). No vitreous cells/haze was noted. Due to his systemic complaints, QuantiFERON Gold, rapid plasma reagin (RPR), HIV Enzyme-linked Immunosorbent Assay and toxoplasmosis serology testing were obtained and found to be normal. An echocardiogram was obtained to assess for possible embolic retinitis and was unremarkable. The retina service was consulted, and retinal imaging was performed. At the level of the two retinal lesions, spectral domain optical coherence tomography (SD-OCT) displayed corresponding hyperreflectivity in the inner retinal layers (Figure 2A,B). Red-free, short wave 488 nm fundus autofluorescence (FAF) as well as infrared imaging highlighted these two lesions (Figure 2 and Figure 3). Fluorescein angiography (FA) revealed normal filling and transit time. Beside the two hypo-autofluorescent lesions FAF showed hyperautofluorescent emboli in the terminal vascular bed of the cilioretinal artery (Figure 3). This latter finding prompted ordering carotid Doppler sonography and subsequently computer tomography angiography, which revealed >90% stenosis within the right carotid bulb primarily related to an atherosclerotic plaque. It was noted that there was passage of only a thin trickle of contrast material with a 1 mm residual lumen diameter. There was a collapse of the distal right internal carotid artery (ICA), related to hemodynamic compromise. Based on these findings, the vascular surgeon performed a successful right carotid endarterectomy with patch graft angioplasty.

**Figure 4:**
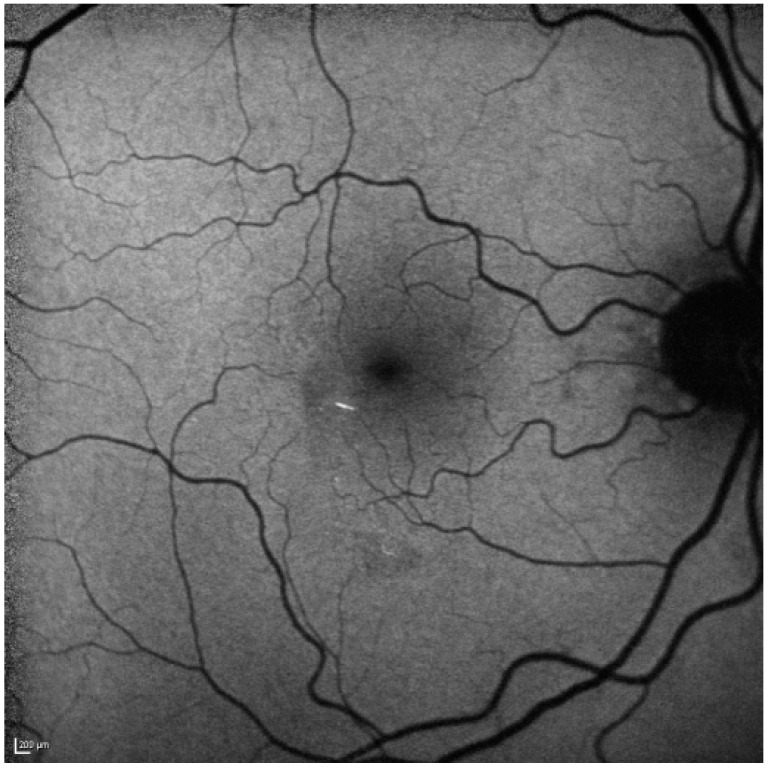
After one month the hypoautofluorescence has faded (as the inner retina atrophies) on short wave blue 488 nm autofluorescence. The hyperautofluorescent emboli are still clearly visible. Scale bar: 200 µm.

One month after presentation, the whitish lesions were nearly invisible. On FAF and IR, the hypoautofluorescent and hyporeflective lesions were still visible but were fading (Figure 2 and Figure 4) and SD-OCT revealed continuous thinning of the retinal nerve fiber layer (RNFL), ganglion cell layer (GCL), inner plexiform layer (IPL) and inner nuclear layer (INL) with a relative thickening of the outer nuclear layer (ONL) (Figure 2). Two months thereafter a subtle hyporeflectivity was still visible on IR, and FAF highlighted the unchanged hyperautofluorescent emboli, while SD-OCT revealed further thinning of respective layers.

## 3. Discussion

Retinal artery occlusions and retinal ischemia can be the first signs of cardiovascular disorders. The case presented here demonstrates that retinal imaging can be crucial for the detection and diagnosis of a cardiovascular disease leading to immediate important therapy.

The retinal blood vessels are responsible for the blood supply of the inner retinal layers, whereas the outer retina is nourished by the choroidal vascular system, with the outer plexiform layer acting as a watershed zone [1,2]. Retinal ischemia due to central, branch, or cilioretinal artery occlusion may present with whitening in the affected non-perfused area, which can be seen in ophthalmoscopy and color photography. The whitening represents swelling and, acutely, is seen as hyperreflectivity throughout all inner retinal layers on SD-OCT. Based on which retinal layers present this localized hyperreflectivity on SD-OCT, the location and level of the thromboembolic event can be presumed [3]. Subsequently, as these areas “die” we see progressive thinning of respective layers over time [4]. The ischemic area that is white and swollen initially scatters and/or absorbs light and therefore is hypoautofluorescent on FAF and is hyporeflective on infrared imaging. As this retina atrophies, these changes fade over time concordant to the subsequent retinal thinning [2,5,6,7]. Although hyporeflectivity on IR is a nonspecific sign for absorbance and/or scattering of light, focal, hyporeflective lesions on IR are, in conjunction with localized hyperreflectivity on SD-OCT and whitening on color photography, indicative of ischemic events. This hyporeflectivity on IR is found irrespective of the severity of the occlusion, *i.e.*, proximal, larger vessel involvement may lead to retinal artery occlusions, affecting all inner retinal layers. Small microvasculature thromboembolic events of the capillary network in turn cause localized ischemic lesions, limited to particular retinal layers [2,3,4,8]. However, the most important finding that led to the diagnosis and subsequently to the work-up and therapy of this patient was the hyperautofluorescent emboli found on FAF. The appearance of such emboli and the usefulness of FAF to detect and illustrate retinal artery occlusions was only noted recently [7]. This previous report assumed that hyperautofluorescence may be present in only certain types of emboli such as calcified emboli [7]. However, in our case the arteriosclerotic plaques in the right carotid bulb visualized by CT angiography and carotid Doppler sonography were described as cholesterol and not calcium containing thromboembolic plaques. In clinical studies of coronary plaques using color fluorescent angioscopy to evaluate fluorescence of the major components of atherosclerotic plaques, it became evident that excitation using short wavelength filters results in an emission spectrum of blue, light blue and white autofluorescence for collagen I, IV and calcium containing plaques, respectively. Cholesterol and cholesteryl esters in turn exhibited a yellow and orange fluorescence [9]. This indicates that the composition of a thromboembolic plaque may be differentiable based on their emission spectrum [9]. The usefulness and applicability of fluorescence to identify retinal emboli need further studies. These findings may then also help guide the differential diagnosis of underlying etiologies [10,11].

In summary, retinal imaging can reveal characteristic findings of retinal ischemia and embolic plaques. This may help to identify patients with cardiovascular diseases in need of life-saving intervention.

## References

[1] Toussaint D., Kuwabara T., Cogan D.G. (1961). Retinal vascular patterns. II. Human retinal vessels studied in three dimensions. Arch. Ophthalmol..

[2] Sarraf D., Rahimy E., Fawzi A.A., Sohn E., Barbazetto I., Zacks D.N., Mittra R.A., Klancnik J.M., Mrejen S., Goldberg N.R. (2013). Paracentral acute middle maculopathy: A new variant of acute macular neuroretinopathy associated with retinal capillary ischemia. JAMA Ophthalmol..

[3] Aleman T.S., Tapino P.J., Brucker A.J. (2012). Evidence of recurrent microvascular occlusions associated with acute branch retinal artery occlusion demonstrated with spectral-domain optical coherence tomography. Retina.

[4] Ritter M., Sacu S., Deak G.G., Kircher K., Sayegh R.G., Pruente C., Schmidt-Erfurth U.M. (2012). *In vivo* identification of alteration of inner neurosensory layers in branch retinal artery occlusion. Br. J. Ophthalmol..

[5] Mathew R., Papavasileiou E., Sivaprasad S. (2010). Autofluorescence and high-definition optical coherence tomography of retinal artery occlusions. Clin. Ophthalmol..

[6] Coady P.A., Cunningham E.T., Vora R.A., McDonald H.R., Johnson R.N., Jumper J.M., Fu A.D., Haug S.J., Williams S.L., Lujan B.J. (2014). Spectral domain optical coherence tomography findings in eyes with acute ischaemic retinal whitening. Br. J. Ophthalmol..

[7] Siddiqui A.A., Paulus Y.M., Scott A.W. (2014). Use of fundus autofluoresecence to evaluate retinal artery occlusion. Retina.

[8] Fawzi A.A., Pappuru R.R., Sarraf D., Le P.P., McCannel C.A., Sobrin L., Goldstein D.A., Honowitz S., Walsh A.C., Sadda S.R. (2012). Acute macular neuroretinopathy: Long-term insights revealed by multimodal imaging. Retina.

[9] Uchida Y., Uchida Y., Kawai S., Kanamaru R., Sugiyama Y., Tomaru T., Maezawa Y., Kameda N. (2010). Detection of vulnerable coronary plaques by color fluorescent angioscopy. JACC Cardiovasc. Imaging.

[10] Ramakrishna G., Malouf J.F., Younge B.R., Connolly H.M., Miller F.A. (2005). Calcific retinal embolism as an indicator of severe unrecognised cardiovascular disease. Heart.

[11] Mughal M.M., Khan M.K., DeMarco J.K., Majid A., Shamoun F., Abela G.S. (2011). Symptomatic and asymptomatic carotid artery plaque. Expert Rev. Cardiovasc. Ther..

